# *Vibrio cholerae* O37: one of the exceptions that prove the rule

**DOI:** 10.1099/mgen.0.000980

**Published:** 2023-04-12

**Authors:** Matthew J. Dorman, Nicholas R. Thomson

**Affiliations:** ^1^​ Wellcome Sanger Institute, Wellcome Genome Campus, Hinxton, CB10 1SA, UK; ^2^​ Churchill College, University of Cambridge, Storey’s Way, Cambridge, CB3 0DS, UK; ^3^​ London School of Hygiene and Tropical Medicine, Keppel St, Bloomsbury, London, WC1E 7HT, UK

**Keywords:** *Vibrio cholerae*, serogroup O37, Sudan, cholera, epidemic

## Abstract

Between 1965 and 1968, outbreaks of cholera in Sudan and former Czechoslovakia provoked considerable public health concern. These still represent important historical events that need to be linked to the growing genomic evidence describing the aetiological agent of cholera, *Vibrio cholerae *. Whilst O1 serogroup *V. cholerae* are canonically associated with epidemic and pandemic cholera, these events were caused by a clone of toxigenic *V. cholerae* O37 that may be more globally distributed than just to Europe and North Africa. Understanding the biology of these non-O1 strains of *V. cholerae* is key to understanding how diseases like cholera continue to be globally important. In this article, we consolidate epidemiological, molecular and genomic descriptions of the bacteria responsible for these outbreaks. We attempt to resolve discrepancies in order to summarize the history and provenance of as many commonly used serogroup O37 strains as possible. Finally, we highlight the potential for whole-genome sequencing of *V. cholerae* O37 isolates from strain collections to shed light on the open questions that we identify.

## Introduction

*Vibrio cholerae* is a Gram-negative bacterium best known for being the aetiological agent of the diarrhoeal disease cholera [1]. The cholera toxin (CT) that causes this diarrhoea is an AB_5_-type toxin, similar to the heat-labile toxin of *Escherichia coli *, and is encoded by the *ctxAB* operon [2, 3]. CT causes the acute watery diarrhoea associated with cholera disease by stimulating adenylate cyclase activity and thereby causing the secretion of chloride ions and water from the gut epithelium into the lumen of the intestine (summarized in [1]). In *V. cholerae *, the *ctxAB* genes are part of a lysogenic bacteriophage called CTX, which infects *V. cholerae* that express the toxin co-regulated pilus (TCP), a pilus which is itself encoded by genes on a mobile pathogenicity island called *Vibrio* pathogenicity island 1, or VPI-I [4, 5]. This example illustrates the fact that horizontal gene transfer (HGT) is a fundamental process in the emergence of toxigenic *V. cholerae* strains. There are multiple allelic variants of the gene encoding the major subunit of TCP, *tcpA*, known to vary between classical and El Tor *V. cholerae *, and which are found on the mobile VPI-I element [6].

The *V. cholerae* species is highly diverse, evidenced by the fact that over 200 serogroups of *V. cholerae* have been described [7, 8]. In spite of this diversity, genomic evidence has shown that global pandemic cholera since 1961 has been caused by a single lineage of toxigenic serogroup O1 *V. cholerae *, dubbed 7PET, for seventh pandemic El Tor lineage [9, 10]. The 7PET lineage also encompasses a sub-lineage that underwent serogroup conversion from O1 to O139, and proceeded to cause epidemics that were restricted in spread to Southeast Asia [10–13]. Current cholera epidemics are caused by serogroup O1 *V. cholerae *, and although serogroup O139 bacteria do not appear to cause epidemics, they do continue to be isolated from clinical cases of disease [14].

A great deal of clinical and public health attention is directed towards *V. cholerae* O1 and O139 [e.g. by the Centers for Disease Control and Prevention (CDC) [15, 16]]. This is logical, given the links between these serogroups and cholera pandemics, and therefore the burden of disease associated with the serogroups [17]. However, at least three examples of large cholera or cholera-like outbreaks being caused by non-O1/O139 *V. cholerae* have been documented, the *V. cholerae* O139 epidemics in Southeast Asia notwithstanding. One such outbreak occurred in former Czechoslovakia during 1965; one in Sudan, 1968, and one on a flight from London to Sydney in 1973 [18, 19]. The London–Sydney outbreak consisted of acute food poisoning contracted during a flight, and a large proportion of those presenting with clinical symptoms were found to be infected with *V. cholerae* of serogroups O7 and O39 in patient specimens, but the toxigenicity of these isolates was not assessed [20].

*V. cholerae* O37 is an important example of non-O1 *V. cholerae* causing large-scale epidemics, but a lack of consolidated information about this serogroup became evident when we researched this subject. In this article, we have sought to collect and review the available bacteriological, molecular and genomic data on *V. cholerae* O37, and to highlight discrepancies in strain details. Several *V. cholerae* O37 have been described in the literature, but their relationship to strains isolated from specific outbreaks is at times unclear. Here, we review these descriptions to place important strains of this serogroup into context, by bringing together molecular, genomic and epidemiological data, along with aggregating important references for the benefit of the community (Table 1). We highlight and attempt to resolve discrepancies amongst these records, to describe the provenance of *V. cholerae* O37 as comprehensively as possible. We emphasize the role for whole-genome sequencing in the future study of this organism, and highlight open questions that genomics might help to resolve. Finally, we stress that just as has been observed for serogroups O1 and O139, serogroup O37 is not itself predictive of cholera outbreaks and epidemics; this serogroup is found in bacteria that are not related to the lineage associated with the outbreaks discussed below.

## Cholera-like outbreaks caused by *V. cholerae* O37

In November 1968, a severe outbreak of gastroenteritis occurred in Idd Eltin, Kassala Province, Sudan [21, 22]. The outbreak was associated with a newly opened well, which had become renowned for curing ‘various incurable maladies’, and around which tens of thousands of people were reported to have gathered without sanitation provisions [22]. Visitors to the well were reported to have bathed in the water, to have smeared mud from the well on their bodies, and to have both consumed well water and taken it from Idd Eltin to elsewhere in the country [22]. The gastroenteritis outbreak was suspected to be cholera, and non-agglutinable *Vibrio* of Heiberg group I were isolated from stool and rectal swab samples [22] (Heiberg groups discriminate biochemically between *V. cholerae* on the basis of sucrose, mannose and arabinose metabolism: group I metabolizes sucrose and mannose, but not arabinose; group II metabolizes sucrose, but neither mannose nor arabinose [23]). Approximately 69 cases were treated in hospitals in a camp hospital established in El Gedaraf District, but there is uncertainty in these and related data [22]. After the well was closed on 14 November 1968, cases of disease declined, and no new cases were reported after 17 November [22]. Whilst the serogroup of the Heiberg I *Vibrio* were determined after the outbreak had occurred, it is now known that the Sudanese outbreak was caused by toxigenic *V. cholerae* of serogroup O37 [18]. The O37 serogroup was formally defined in 1970 [24]. The type strain for the serogroup, named 1322–69, was isolated in India during 1969 and – importantly – is toxigenic; this will discussed in more detail later in this article (Table 1) [25–27].

**Table 1.** *
V. cholerae* O37 strains in common use and of historical importance

Strain name	Details	Select references
*Serogroup O37, Sudan lineage or closely related to Sudan lineage strains*		
V52	Sudan, 1968. Toxigenic, harbours VPI-I and VPI-II. *hapR* mutant	[4, 37, 63*]
ATCC 25872 (280 NAG, possibly NCV 10125)	Czechoslovakia, 1965. Toxigenic, harbours VPI-I. Wild-type for *hapR*. Recently sequenced	[4, 28, 63, 79]
S-21	Sudan, 1968. Toxigenic, harbours VPI-I. Molecular data indicate that this is closely related to V52 and ATCC 25872	[4, 50, 51]
CO130	India, 1993. Toxigenic. Lacks VPI-II	[40, 41], discrepancies with the report of [42]
1322–69	India, 1969. Toxigenic. Type strain for serogroup O37. Closely related to El Tor and classical lineage isolates	[25–27, 49]
S7	Clinical isolate, Sudan, 1968. Toxigenic. Originated from CDC. Original source of *ctxB9* sequence	[22, 35, 36, 56, 57]
G12R	Clinical isolate, Sudan, 1968. Toxigenic. Originated from CDC	[22, 35, 37*]
*Putatively serogroup O37 and closely related to Sudan lineage*		
M1618 (N1, V523)	Environmental isolate. Australia, 1977. Toxigenic, harbours VPI-I. Has not been serogrouped	[50]
*Serogroup O37, distantly related to Sudan lineage strains, or provenance uncertain*		
MZO-3	Clinical isolate, Bangladesh, 2001. Non-toxigenic. Not part of the O37/Sudan lineage	[54]
SG8	Clinical isolate, India, 1992–1993. Non-toxigenic. Lacks IS*1004*	[35]
151	Mexico. Non-toxigenic, but harbours a pre-CTX like element. Ambiguities relating to serogroup and year of origin	[40, 43, 47, 48]
VO7	India, 1988. Non-toxigenic. Harbours VSP-I	[47, 48]
CO476	India, 1994. Non-toxigenic	[48]
ATCC 25873 (281 NAG)	Czechoslovakia, 1965. Serogroup O37	[28, 29, 32]

*Although ref. 37 is the original citation for certain Sudanese strains, cited throughout the literature, we have not been able to obtain a copy of this manuscript to review ourselves

The nature of the next example of an O37 outbreak is not so well recorded. During 1965, a foodborne outbreak of gastroenteritis occurred amongst individuals at an automobile training centre in Czechoslovakia [28]. Patients were described as producing stool that contained neither blood nor mucus [28]. The isolate linked to this outbreak that was deposited in the American Type Culture Collection (ATCC; accession number ATCC 25872) is recorded as having being taken from a ‘patient with clinical cholera’ from this outbreak. The outbreak itself was ascribed to the consumption of potatoes contaminated with a *Vibrio* sp. named NCV 10125 [28].

It should be noted that there is some ambiguity as to whether NCV 10125 is the same as the ATCC 25872 strain deposited by Felsenfeld (also named ‘280 NAG’ in ATCC records). NCV 10125 was originally classified as a Heiberg group II *Vibrio* [28], and the description of 280 NAG is that of a Heiberg group I strain [29]. Two other *V. cholerae* were deposited simultaneously by Felsenfeld into ATCC [29]: ATCC 25873 (also named ‘281 NAG’) and ATCC 25874 (‘123 NAG’). Like ATCC 25872, both were originally obtained from Aldová [30, 31]. ATCC 25873 is known to be of serogroup O37 [32], suggesting that ATCC 25872 and ATCC 25873 are from the same Czechoslovakian outbreak. However, molecular data from rRNA intergenic spacer region (ISR) sequencing show that ATCC 25872 and ATCC 25874 are not identical to one another (99.8 % sequence similarity at the ISR-2 region, and 99.5 % similarity at ISR-3) [33]. Adding to this uncertainty, some articles have also described the Czechoslovakian outbreak as being caused by serogroup O5 *V. cholerae* [18, 34]. However, we have been unable to locate the original data that support that statement, and there is a body of evidence, discussed below, which indicates that both the Sudanese and Czechoslovakian outbreaks were in fact caused by *V. cholerae* O37.

## The relationship between *V. cholerae* O37 and pandemic *V. cholerae* O1

The relationship between *V. cholerae* O37 and pandemic lineage *V. cholerae* O1 was first investigated using several molecular techniques, which will be discussed in chronological order here. In 1996, Bik *et al*. used IS*1004* molecular fingerprinting to characterize a number of *V. cholerae *, including two Sudanese isolates from 1968: S7 and G12R [35–37]. Both of these isolates were toxigenic and were of the same IS*1004* molecular fingerprint type, C3 [35]. Moreover, these were found to be highly similar to the C1 fingerprint, associated with classical *V. cholerae* strains from the sixth pandemic [35]. A third serogroup O37 isolate, SG8, was also included in this study. SG8 was a clinical isolate obtained in India between 1992 and 1993, but was non-toxigenic, and did not possess IS*1004* [35]. The authors commented that just as strains that were closely genetically related can express different serogroups, referring to O1 and O139 *V. cholerae *, so too could they identify strains that expressed the same serogroup but were distantly related to one another genetically, referring to SG8 and S7/G12R (Table 1) [35]. Their comment that the study ‘exemplifies the pitfalls of using phenotypic methods to discriminate *V. cholerae* strains’ [35] seems particularly apposite to the current narrative.

The theme that *V. cholerae* serogrouping as O37 can be both members of the same lineage as well as being unrelated to that lineage(s) was subsequently reiterated [25]. In this study, electrophoretic typing (ET) by multi-locus enzyme electrophoresis (MLEE) rather than molecular fingerprinting was used to classify 254 isolates from Mexico and Guatemala, plus 143 of the *V. cholerae* serogrouping reference strains [38], into 279 electrophoretic types. The authors noted that ‘there are two examples of epidemic *V. cholerae* expressing a non-O1 antigen’ [25], referring to *V. cholerae* O139 and the Sudanese outbreak of *V. cholerae* O37 [25]. The authors included four *V. cholerae* O37 in their study, including three isolates from Mexico and Guatemala, as well as the O37 serotyping reference strain that had been isolated clinically in India during 1969. They assigned this strain to ET5 and demonstrated that it harboured *ctxA* (encoding the cholera toxin A subunit), stating that it ‘presumably represents the same clone’ as that which caused the Sudanese outbreak [25]. An O37 *V. cholerae* isolate from Guatemala was assigned to ET6, and despite being closely related to ET5 by MLEE, this isolate lacked *ctxA* [25]. The authors speculated that this Guatemalan isolate may ‘represent an offshoot […] in which the CTX genetic element has been deleted’ [25]. The two remaining *V. cholerae* O37 in this study, cultured from well water in Campeche (Mexico), were of ETs 75 and 149, and by this measure were distantly related to the toxigenic *V. cholerae* O37 [25].

Beltrán *et al.* also found that the O37 reference isolate was of the same ET as the non-toxigenic serogroup O102 reference isolate, obtained from PR China in 1988 [38]. However, the authors acknowledged that sequence data would be required in addition to MLEE data to understand the clonal relationships of *V. cholerae *, and to account for the possible role of recombination amongst these bacteria [39]. The authors concluded that the O37 clone to which Sudanese and Indian isolates belonged was circulating in those regions during 1968–1969 [25]. As discussed elsewhere in this review, other molecular evidence indicates that the Czechoslovakian outbreak isolates are likely to belong to this clone.

## Application of single-locus and multi-locus sequencing approaches to *V. cholerae* O37

Boyd and colleagues found that CO130, a toxigenic *V. cholerae* O37 isolated from the environment in India, 1993, was closely related to V52, based on phylogenetic analysis of the single genes *mdh*, *orfU* and *zot* [40]. The authors included *mdh* as a commonly used chromosomal locus for phylogenetic analysis of other bacteria, to contrast with the sequence alignment of *orfU* and *zot*, both encoded by the CTX bacteriophage [40]. Subsequently, it was shown that V52 and CO130 were closely related to one another chromosomally, but that V52 had a classical-like TcpA and CO130 had an El Tor TcpA (Table 1) [41]. It is unclear whether the environmentally obtained CO130 first described in [40] is the same O37 isolate reported in [42], since although the date and place of origin as well as serogroup are the same, all of the isolates reported by Mukhopadhyay and colleagues were of clinical origin from hospital surveillance [42]. There are also discrepancies between the serogroup O37 isolate named 151, from Mexico and isolated in 1993, described in [40], whose authors cite the original report of its isolation [43]. However, in that study, 151 is reported to be serogroup O34 and isolated in 1986 [43]. Boyd *et al.* also refer separately in their report to this isolate being of serogroup O11 [40]. It is possible that these discrepancies might reflect the use of alternative serogrouping schemes [27, 44–46].

Phylogenetic analysis of the sequences of two housekeeping genes, *mdh* and *groEL*, has been used to infer the evolutionary relationships of several *V. cholerae* that were obtained from a wide variety of geographical locations [47]. Among the strains in this study were seven serogroup O37 isolates: V52 and V53 from the Sudanese outbreak, CO130 and four non-toxigenic isolates – VO7 (isolated in India during 1988), CO476 (India, 1994), the aforementioned SG8, and strain 151, which was described as being from Mexico, 1998, and as being non-toxigenic but harbouring CTX, consistent with Boyd and colleagues’ finding that 151 harboured a ‘pre-CTX’ like element but lacked *ctxAB* [40, 47]. Previous results showing the close relationship between these Sudanese isolates and pandemic *V. cholerae* were recapitulated [41, 47]. CO130 was shown to be closely related to the Sudanese outbreak strains, although unlike the Sudanese strains it was found to lack the VPI-II pathogenicity island [47] (Table 1). The other four *V. cholerae* O37 were much more distantly related [47]. The non-toxigenic isolate VO7 was unusual because it harboured a pathogenicity island (VSP-I) thought to be found only in seventh pandemic *V. cholerae *, but it clustered with an environmental O1 from Louisiana, 1979, and was not related to pandemic *V. cholerae* O1 or O139 [47].

Reen and Boyd applied another molecular approach – polymerase chain reaction single-strand conformation polymorphism analysis (PCR-SSCP) – to the differentiation of epidemic and non-epidemic *V. cholerae* [48]. Their work included six *V. cholerae* O37 – toxigenic V52 and V53, and four non-toxigenic isolates, SG8 and VO7, together with strain 151 from Mexico (metadata as reported in [47]) and CO476 from India. Once again, their data showed that V52 and V53 displayed the same PCR-SSCP profile as the classical and El Tor *V. cholerae* O1 associated with pandemics, and the four non-toxigenic isolates displayed dissimilar profiles [48]. This is consistent with a subset of *V. cholerae* O37 being members of a toxigenic *V. cholerae* lineage.

Evidence that the 1322–69 serogroup reference strain is part of the same O37 lineage was presented by Li and colleagues, who used IS*1004* fingerprinting to find that this strain was most similar to toxigenic serogroup O1 classical biotype isolates [49]. Their results were concordant with previous findings, including those that showed the close relationship between classical and O37 isolates [25, 35]. A subsequent study using whole-genome mapping data confirmed that the Sakazaki O37 reference strain 1322–69 is toxigenic and clusters near to pandemic El Tor and classical strains [26].

Data from multi-locus sequence typing (MLST) further corroborate this understanding of *V. cholerae* O37 population structure [50]. The authors suggested that V52 and S-21 were likely to be from the same outbreak; both were isolated in 1968 from Sudan [50]. They also found that ATCC 25872 was identical to V52 and S-21 by MLST. This is fully consistent with both the Czechoslovakian and Sudanese outbreaks of 1965 and 1968 being caused by the same lineage of *V. cholerae* O37, and was corroborated by a subsequent MLST study that showed ATCC 25872 and S-21 to form a clonal complex with pandemic serogroup O1 El Tor strains [51]. Surprisingly, they also found that a non-O1/O139 environmental isolate M1618 (also named N1 and V523; Table 1) obtained in Australia during 1977 was identical to the Sudan clone, and was both toxigenic and harboured VPI-I [50]. This, together with the finding in 1999 that a non-toxigenic *V. cholerae* O37 from Guatemala was closely related to O37 lineage isolates [25], strongly suggests that this O37 lineage is globally distributed.

## Reconciling genomic data with molecular observations

**Fig. 1.**
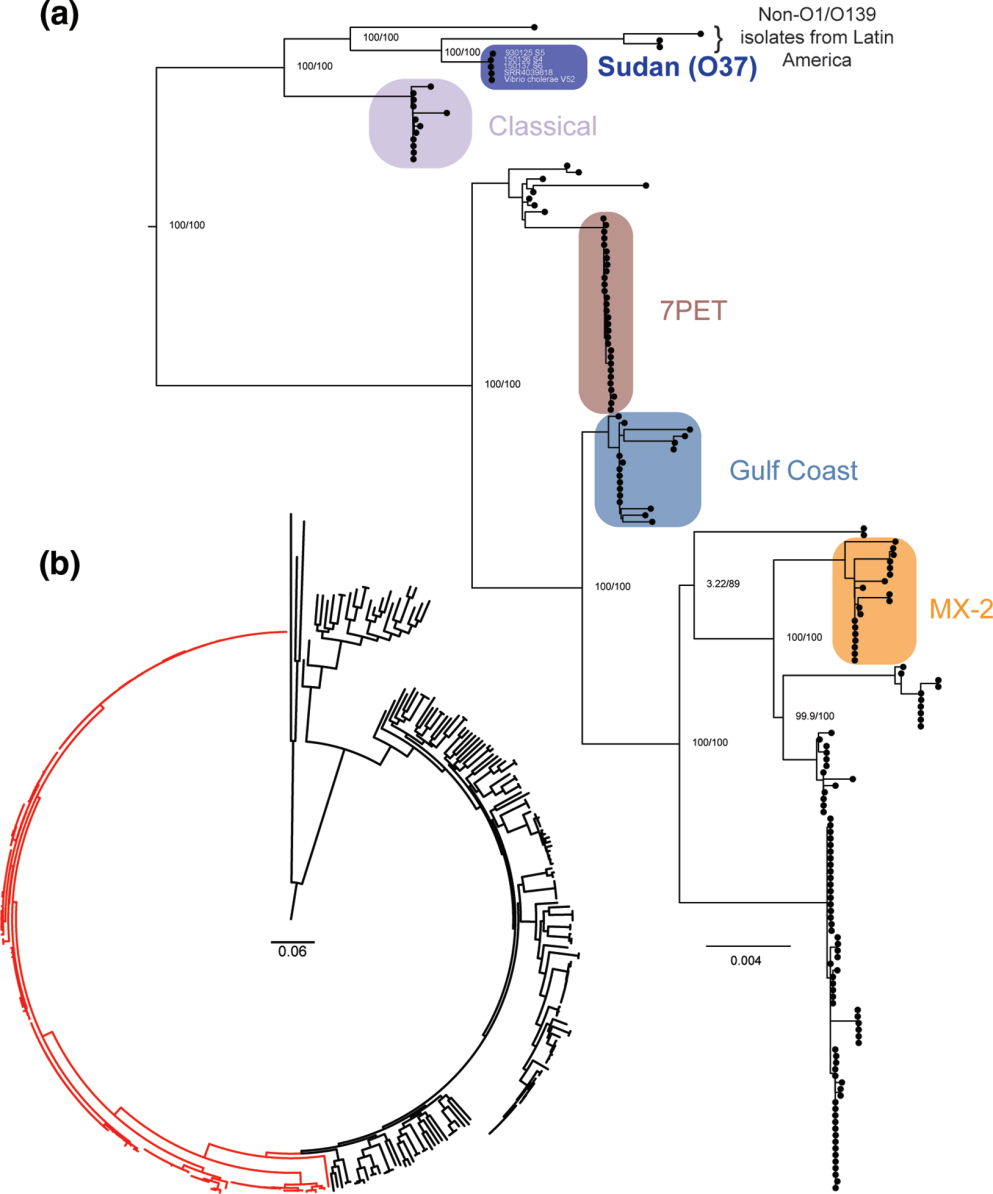
The position of the O37 Sudan lineage within a *V. cholerae* phylogenetic tree. (**a**) A phylogenetic sub-tree comprising 177 isolates of *V. cholerae *, in which serogroup O37 isolates belonging to the Sudan lineage are highlighted to illustrate their relationship to other major lineages of the species [9]. Isolate names can be matched to supplementary tables in [9, 76]. (**b**) The complete phylogeny of 383 isolates, from which the sub-tree in (**a**) was extracted, indicated in red. The tree used to construct this figure has been previously published under a CC-BY Open Access licence as part of [76], and can be downloaded from the CC-BY Figshare repository linked to that paper [80]. Scale bar, substitutions per variable site. Ultrafast rapid bootstrap approximation and approximate likelihood ratio test values for major nodes are reported (see original article for details of phylogeny construction). Figure prepared using Figtree v1.4.3 and Adobe Illustrator CC v23.1.1.

Genomic analysis has provided additional evidence with which to corroborate the phylogenetic inferences drawn about the relationship of the O37 lineage to pandemic lineage *V. cholerae *, and the fact that other *V. cholerae* O37 are not related to this lineage. In one of the first whole-genome analyses of *V. cholerae *, Chun and colleagues showed V52 to be part of PG-2, a group of strains that were phylogenetically closely related to classical *V. cholerae* [52]. This result was recapitulated by Mutreja *et al.*, who assigned V52 to lineage L7, a lineage that was closely related to the L1 lineage of classical biotype *V. cholerae* [10]. V52 has since been resequenced [53], and the phylogenetic position of that isolate remains the same as the original V52 sequence (Fig. 1).

Domman and colleagues performed a phylogenetic analysis that included the genomes of three additional Sudanese *V. cholerae* O37 isolates from 1969 [9]. All three isolates were toxigenic, harboured VPI-I and VPI-II, and formed a phylogenetic cluster with V52, fully consistent with the Sudanese outbreak being caused by a clone of *V. cholerae* O37. Moreover, this lineage was itself closely related to the classical lineage of *V. cholerae* O1 [9], illustrated here in Fig. 1. With this observation in mind, and to ensure consistency with contemporary genomic descriptions of *V. cholerae* population structure, we propose that the toxigenic *V. cholerae* O37 clone found in Sudan and by extrapolation from the studies discussed above, in Czechoslovakia, Australia and possibly Guatemala, be referred to as the Sudan lineage of *V. cholerae *.

Genomic data also emphasize the fact that serogroup O37 is not unique to the Sudan lineage. For instance, the serogroup O37 strain MZO-3 was isolated in 2001 and first described in 2005 [54]. This strain is non-toxigenic, though it does harbour pathogenicity islands, including VSP-II [54]. MZO-3 was sequenced by Chun *et al*. [52], who confirmed that this did not cluster with V52, an observation recapitulated in other studies [9]. This is fully consistent with the studies discussed previously in this article, which report *V. cholerae* O37 that are non-toxigenic and distantly related to V52, ATCC 25872, or other related isolates (e.g. [25, 35]). There have been recent clinical reports of infection caused by non-toxigenic *V. cholerae* O37 that do not harbour VPI-I, but are cytotoxic in *in vitro* models [55]. Even in the absence of phylogenetic or genomic data, it is very unlikely that these bacteria are members of the Sudan lineage.

A unique aspect of *V. cholerae* O37 is the *ctxB* allele found in toxigenic members of the Sudan lineage, *ctxB9*. Yamamoto and colleagues presented evidence in 1983 that the Sudanese strain S7 is toxigenic [56], and subsequently described the sequence differences between *ctxB* from S7 and classical *V. cholerae* [57]. This is particularly relevant because the *tcpA* allele harboured by *V. cholerae* O37 was that associated with classical biotype *V. cholerae* rather than El Tor [41]. To our knowledge, *V. cholerae* have not yet been identified that harbour *ctxB9* other than Sudan lineage *V. cholerae* O37 [9, 58]. Although it might be tempting to use *ctxB9* as a marker of *V. cholerae* O37, it is important to recall that not all *V. cholerae* O37 are toxigenic [25, 38], and that because *ctxB* is encoded by a mobilizable prophage, we cannot exclude the possibility that this gene is present in other *V. cholerae* just because we have only found *ctxB9* in members of this lineage to date.

## The contribution of *V. cholerae* O37 to basic science

In addition to their historical and epidemiological significance, the laboratory strains derived from both the Sudanese and Czechoslovakian outbreaks, V52 and ATCC 25872 respectively (Table 1) [29, 37], are important reference strains used in *V. cholerae* experimental research. For instance, ATCC 25872 was used in the original characterization of the TCP-encoding VPI element (now renamed VPI-I), work which showed that *V. cholerae* O37 can harbour the VPI-I genomic island encoding the CTX bacteriophage receptor and thereby become toxigenic through lysogenic conversion [4]. V52 has become a useful model organism for the study of a multitude of additional traits in *V. cholerae *. For example, unlike laboratory strains of *V. cholerae* O1, V52 and ATCC 25872 constitutively express type VI secretion systems (T6SSs) and are used as control strains for the study of T6SS activity in this species [59–61]. Although V52 is not an appropriate strain to use for the study of quorum sensing, due to a mutation in *hapR* [62], ATCC 25872 does not have this mutation and is quorum sensing proficient [63]. V52 has also been used for studying the *V. cholerae* acetylome [64], and in the study of alternative sigma factors and their regulons in *V. cholerae* [65]. The genome of V52 was sequenced in 2009 [52], and this isolate’s genome continues to be an important research focus, including in recent work reporting that positive selection may be occurring in V52 relative to other *V. cholerae* [66].

*V. cholerae* O37 have also been used as important models for studying the roles of recombination and horizontal gene transfer in serogroup conversion in this species. Once it was shown that certain strains of *V. cholerae* O37 were closely related to classical *V. cholerae* O1 (discussed more deeply elsewhere in this review), the similarity of the sequence of DNA flanking the O-antigen locus in *V. cholerae* O1 and *V. cholerae* O37 led to the hypothesis that homologous recombination had caused the genes conferring an O1 genotype to have been exchanged with those encoding the O37 serogroup, causing seroconversion [49]. This was of particular relevance because similar recombination events had been suggested to have enabled seroconversion of *V. cholerae* O1 to O139 [13, 49, 67, 68]. Subsequently, Blokesch and Schoolnik demonstrated that genomic DNA prepared from O37 serogroup strain ATCC 25872 could be used to transform naturally competent *V. cholerae* O1 and convert them to serogroup O37 [32]. Their work also showed that O1-to-O139 seroconversion could occur in the same way [32], suggesting a general mechanism by which *V. cholerae* can participate in horizontal gene transfer via natural competence and undergo serogroup exchange. Other work has also characterized the horizontal transfer of genes encoding the *V. cholerae* O-antigen, including O1 and O37, as well as the DNA sequence junctions implicated in the recombination and exchange events [69].

## Conclusions, open questions and future directions

Collectively, the studies reviewed in this article indicate that during the 1960s a toxigenic clone of *V. cholerae* O37 was present in Sudan, former Czechoslovakia and India. This clone may also have been present in Guatemala prior to 1993 [25], and in Australia during 1977 [50]. In order to ensure consistency with published phylogenetic studies, and in recognition of the acute outbreak of disease that occurred during 1968, we have proposed that this clone of *V. cholerae* O37 be referred to as the Sudan lineage (Fig. 1) to differentiate it from pandemic and other local lineages, and because of its historical importance – both to epidemiologically relevant outbreaks of disease and to basic science. However, it is equally important to recall that throughout this review, *V. cholerae* O37, which do not form part of the Sudan lineage, have been a recurring theme. This is reminiscent of two other examples from *V. cholerae* history – firstly, the fact that *V. cholerae* O139 can be isolated both clinically and environmentally that are not members of the O139 sub-lineage of 7PET (e.g. [70–72]), and secondly, that not all toxigenic *V. cholerae* O1 are part of the pandemic lineages (e.g. [9, 73, 74]). The fact that seroconversion can occur between *V. cholerae* O1 and O139, just as it can occur between O1 and O37, underscores the importance of stating that serogroup alone is not a reliable predictor of the relative risk posed by a *V. cholerae* isolate to public health.

Not all cases of disease caused by *V. cholerae* are due to infection with toxigenic bacteria, and alternative virulence determinants such as type 3 secretion systems can contribute to disease (e.g. [54, 69, 75]). However, genomic work in Latin America proposed that *V. cholerae* O1 can cause three patterns of disease – sporadic cases or outbreaks often linked to foodborne infection, local epidemics over sustained periods of time and pandemic cholera [9]. These patterns of disease were described using data from non-7PET *V. cholerae* O1 [9], and more recent work has provided further evidence that pandemic and non-pandemic *V. cholerae* display different dynamics during the progression of a cholera epidemic [76]. However, given that the Sudan lineage has been associated with several historical epidemics and appears to be globally distributed, this lineage of serogroup O37 *V. cholerae* meets the criteria of the first pattern of disease – i.e. a lineage linked to sporadic and localized outbreaks of cholera [9]. Thus, as alluded to in the title of the article, the Sudan lineage is a non-O1 non-pandemic exception that proves the rule of at least one of these patterns of disease.

An obvious gap in our knowledge about *V. cholerae* O37 is the paucity of genome sequences of bacteria from this lineage. Understanding commonly used reference strains in the context of a species allows researchers to determine whether observations in a laboratory strain explain the species, a subset of the species, or just that strain in general [77, 78]. Although some additional Sudanese genomes were published in 2017 [9], we still lack genome sequences for many of the interesting strains that have been discussed. It would be similarly important to sequence the geographically dispersed strains that appear to be part of this O37 lineage, since until proven phylogenetically, their membership remains speculative. However, once sequenced, standard phylogenetic approaches will enable the reconstruction of the history of this lineage, and have the potential to develop our understanding of how this lineage might have spread globally, from where it may have originated and whether there is a risk that it may cause sporadic outbreaks again in the future.
